# Construction and Analysis of an Enzyme-Constrained Metabolic Model of *Corynebacterium glutamicum*

**DOI:** 10.3390/biom12101499

**Published:** 2022-10-17

**Authors:** Jinhui Niu, Zhitao Mao, Yufeng Mao, Ke Wu, Zhenkun Shi, Qianqian Yuan, Jingyi Cai, Hongwu Ma

**Affiliations:** 1 School of Life Sciences, Division of Life Sciences and Medicine, University of Science and Technology of China, Hefei 230026, China; 2 Biodesign Center, Tianjin Institute of Industrial Biotechnology, Chinese Academy of Sciences, Tianjin 300308, China; 3 National Technology Innovation Center of Synthetic Biology, Tianjin 300308, China

**Keywords:** enzyme-constrained model, *Corynebacterium glutamicum*, metabolic engineering

## Abstract

The genome-scale metabolic model (GEM) is a powerful tool for interpreting and predicting cellular phenotypes under various environmental and genetic perturbations. However, GEM only considers stoichiometric constraints, and the simulated growth and product yield values will show a monotonic linear increase with increasing substrate uptake rate, which deviates from the experimentally measured values. Recently, the integration of enzymatic constraints into stoichiometry-based GEMs was proven to be effective in making novel discoveries and predicting new engineering targets. Here, we present the first genome-scale enzyme-constrained model (ecCGL1) for *Corynebacterium glutamicum* reconstructed by integrating enzyme kinetic data from various sources using a ECMpy workflow based on the high-quality GEM of *C. glutamicum* (obtained by modifying the iCW773 model). The enzyme-constrained model improved the prediction of phenotypes and simulated overflow metabolism, while also recapitulating the trade-off between biomass yield and enzyme usage efficiency. Finally, we used the ecCGL1 to identify several gene modification targets for l-lysine production, most of which agree with previously reported genes. This study shows that incorporating enzyme kinetic information into the GEM enhances the cellular phenotypes prediction of *C. glutamicum*, which can help identify key enzymes and thus provide reliable guidance for metabolic engineering.

## 1. Introduction

*Corynebacterium glutamicum* is widely known as an excellent producer of amino acids [1]. Recent advances in metabolic engineering and synthetic biology have expanded the scope of chemicals that can be produced from *C. glutamicum*, but it remains difficult to synthesize these compounds on an industrially relevant scale [2]. Genome-scale metabolic models (GEMs) are a proven tool for the prediction of cellular behavior and the discovery of potential engineering targets [3]. Several GEMs of *C. glutamicum* have been developed (Figure S1), and used to guide the production of high-value compounds such as glutaric acid [4], anthocyanins [5] and l-glutamate family amino acids [6]. The most widely used model of *C. glutamicum* is iCW773, constructed in 2017 [7], which accurately predicts the growth of cells cultured under different conditions. Although the quality of *C. glutamicum* models has improved in the last decade, they mostly only consider reaction stoichiometries and do not accurately depict the real situation inside the cell [8]. For example, metabolic overflow is a phenomenon in which incomplete oxidation of glucose to ethanol or acetate occurs in microorganisms in the presence of sufficient substrate, which has been recognized for a long time and frequently occurs in microbial cultures [9]. It has been shown that the limitation of intracellular protein resources is the cause of the metabolic overflow phenomenon [10], which cannot be properly explained by models only considering reaction stoichiometries.

With the accumulation of enzyme kinetic data and the availability of high-throughput omics data, it has become possible to integrate these data into models to add constraints on individual reactions or aggregate constraints on enzyme resources [11,12]. In 2007, Zhang et al. constructed the FBAwMC model by introducing both a crowding coefficient and cell volume constraint to limit the space occupied by enzymes [13]. Subsequently, the researchers developed other protein resource integration methods [14], which are referred to as enzyme-constrained genome-scale models (ecGEMs), including MOMENT [15], GECKO [16], AutoPACMEN [17] and ECMpy [18]. The GECKO method was reported in 2017 and was applied to construct an enzyme-constrained model of *Saccharomyces cerevisiae* by adding many pseudo-metabolites to represent enzyme utilization and including *k*_cat_ values to expand the stoichiometric matrix. Notably, this enzyme-constrained model could accurately predict several metabolic phenotypes. Recently, Domenzain et al. upgraded GECKO to 2.0 to enhance models with enzyme and proteomics constraints for any organism with a compatible GEM reconstruction, which also proposed an automated calibration process for enzyme kinetic parameters and developed conventional algorithms based on ecGEMs (e.g., flux variability analysis) [19]. Inspired by MOMENT and GECKO, in 2020, Bekiaris et al. proposed a simpler method for enzyme-constrained model construction, called AutoPACMEN [17], which could automatically download kinetic parameters of enzymes from the BRENDA [20] and SABIO-RK [21] databases. It was used to construct an enzyme-constrained model of *Escherichia coli*, which only introduced one pseudo-reaction and pseudo-metabolite into the stoichiometric matrix. Different from GECKO and AutoPACMEN, ECMpy simply adds a constraint on the total amount of enzymes and does not require modification of the stoichiometric matrix, while providing higher prediction accuracy for the simulation of the *E. coli* growth rate [18]. Currently, ecGEMs have been constructed for many species, such as *E. coli* [18,22], *S. cerevisiae* [16], *Yarrowia lipolytica* [19], *Aspergillus niger* [23], and *Bacillus subtilis* [24].

In the enzyme-constrained models, *k*_cat_ and molecular weight (MW) of an enzyme set constraints on the fluxes of the reactions catalyzed by that enzyme. Previous studies have made efforts to automatically acquire *k*_cat_ values from databases and fill missing values using methods like machine learning [25]. In contrast, few studies have paid attention to molecular weight. It may seem straightforward to obtain the MW of a protein from databases like UniProt. However, the MW values from these databases are for monomers and many enzymes contain two or more subunits. For example, 6-phosphogluconate dehydrogenase encoded by gene *Cgl1452* is a homodimer and, therefore, the MW is 105.2 kDa instead of 52.6.2 kDa for the monomer. There are also many enzymes consisting of subunits encoded by different genes which are represented as ‘and’ GPR relationship in GEMs. However, what is missing in the GPR relationships is the number of each subunit in the protein complex. For example, Succinyl-CoA synthetase is a heterotetramer containing two alpha subunits (encoded by *Cgl2565* in *C. glutamicum* with an MW of 30.26 kDa) and two beta subunits (encoded by *Cgl2566* with an MW of 41.76 kDa). Therefore, the MW of this enzyme complex should be 144.04 kDa (2 × 30.26 + 2 × 41.76) instead of 72.02 kDa. Such quantitative information on enzyme subunit composition is difficult to obtain from databases and often missing in the published GEMs, leading to incorrect MW values which affect the prediction accuracy of enzyme-constrained models.

In this study, we first systematically corrected the GPR relationships in the iCW773 model based on the GPRuler tool [26] and protein homology similarity comparisons, and extend the GPRuler tool to allow access to the quantitative subunit composition of each protein in the model. Then, we gathered the enzyme kinetics data of *C. glutamicum* using AutoPACMEN and constructed the enzyme-constrained model ecCGL1 based on the ECMpy workflow. We further comprehensively evaluated ecCGL1 and confirmed that it had a better prediction accuracy of phenotypes than iCW773^R^ (Revised iCW773) and could simulate a variety of biological phenomena. Finally, we applied ecCGL1 to metabolic engineering and discovered potential targets for increasing the production of l-lysine.

## 2. Materials and Methods

### 2.1. Model Calibration

We obtained the iCW773 model, which has 773 genes, 1207 reactions, and 950 metabolites, from the supplemental data of Zhang et al. [9], and converted it to XML format. To meet the requirements of the AutoPACMEN and ECMpy processes for metabolic network format input, we modified the gene, reaction and metabolite information in the model as follows:

(1) Metabolite correction: ‘(e)’ to ‘_e’, ‘-D’ to ‘__D’, ‘-L’ to ‘__L’, ‘-R’ to ‘__R’ and other ‘-’ to ‘_’.
(2) Reaction correction: ‘-’ to ‘__’ in reactions beginning with ‘EX’ and ‘-’ to ‘__’ in other reactions.
(3) Adding UniProt ID information to the annotation, which is the basis for obtaining kinetic parameters.

### 2.2. Correction of GPR Relationship

We found some errors in the GPR relationships in iCW773 during the analysis and corrected these errors using two methods. First, the modified GPRuler tool was used to identify more “and” relationships. The GPRuler tool identifies the ‘and‘ relationships based on the protein complex information extracted from databases such as UniProt [27] and Complex Portal [28]. However, the original terms (‘subunit’ and ‘chain’) used to identify complexes in the GPRuler tool were extracted based on human and yeast protein description information and did not cover all *C. glutamicum* protein complexes. For example, the protein name of P06557 (encoding by *Cgl3029*) is Anthranilate synthase component 1, which will not be identified as a subunit forming an ‘and‘ relationship with another subunit using the original terms. Therefore, we updated the terms by carefully checking the words used in UniProt to describe possible protein complex formation (e.g., ‘component’, ‘binding protein’, and ‘assembly factor’, etc. see Table S1 for the full list) to obtain more ‘and‘ relationships in *C. glutamicum*. We also simplified the GPRuler tool process by parsing UniProt data directly to obtain the corresponding GPR relationships without running the processes for gene identification, reaction identification, and gene filter.

We also observed that some ‘and‘ relationships in iCW773 were not identified by the GPRuler tool and could be wrong. To address this, we developed a semi-automated process based on protein similarity to determine the correct relationship. We calculated the protein sequence similarity for the remaining ‘and’ relationships in iCW773 and revised the relationship to ‘or‘ if similarity exists between protein sequences as proteins with similarity are more likely to be isoenzymes rather than forming protein complexes. We then manually checked the gene annotation information in databases (BioCyc [29] and KEGG [30]) to ensure the correction is right.

### 2.3. Acquisition of Quantitative Subunit Composition

As discussed in the Introduction section, quantitative subunit information of an enzyme is essential to correctly determine its MW but is missing in the GPR relationships in the models. We have manually collected the subunit number of each protein in our previous approach for constructing the enzyme-constrained model of *E. coli* eciML1515 [18]. Here, we used a new automatic method to acquire the quantitative subunit information by extending the GPRuler tool to resolve the subunit number of a protein based on information in the ‘Interaction information’ section in UniProt. For example, Q8NMK2 is described in UniProt as ‘Homodimer’, so its subunit number is 2. We created a word list to parse the description information (e.g., Homodimer; Heterotrimer; Tetramer of two alpha and two beta chains) and translated them into corresponding subunit numbers (Table S2).

### 2.4. Construction of ecCGL1

**Figure 1:**
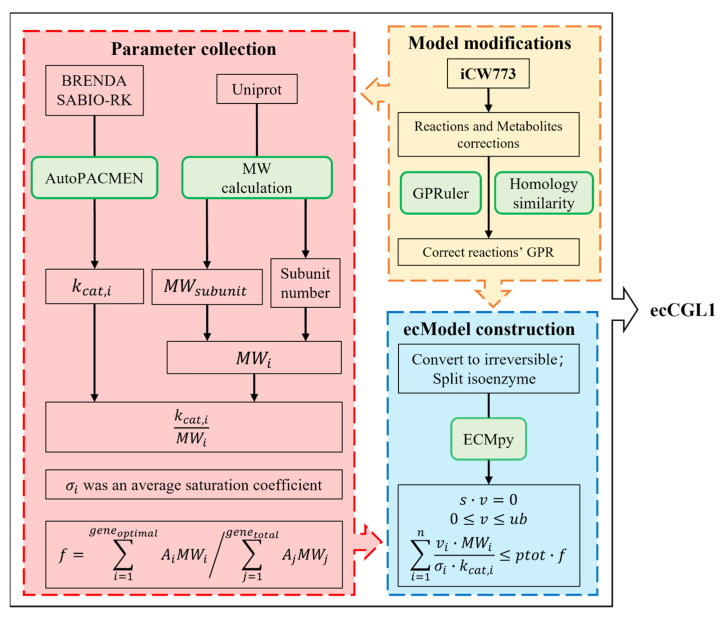
Workflow for the construction of ecCGL1.

After model correction, we split the reversible reactions in the model into two irreversible reactions because of different *k*_cat_ values for different catalytic directions. We also split the isozyme-catalyzed reaction into multiple individual enzyme-catalyzed reactions. Then, the molecular weight for each protein monomer was obtained automatically from Uniprot using AutoPACMEN and combined with the protein subunit composition data obtained in Section 2.3 to calculate the molecular weight for each enzyme using equation $MW=\sum_{j=1}^{m}N_{j}*MW_{j}$, where m is the number of different subunits in the enzyme complex and N_j_ is the number of jth subunits in the complex. We further obtained the kinetic parameters of the enzymes mainly from BRENDA and SABIO-RK, using AutoPACMEN. In addition, we calculated the mass fraction of total cellular enzymes (Equation (4)) using published RNA-Seq data of wild-type *C. glutamicum* ATCC 13032 grown on glucose [31,32]. Finally, we used the ECMpy process to construct ecCGL1 (see Figure 1 for details), which can be mathematically represented as follows:

(1) $$Z=\max\left\{C^{T}*v\right\}$$

(2) S∗v=0

(3) lb≤v≤ub

(4) $$f=\overset{n\left(gene_{model}\right)}{\underset{i=1}{\sum }}A_{i}MW_{i}/\overset{n(gene_{total})}{\underset{j=1}{\sum }}A_{j}MW_{j}$$

(5) $$\overset{n}{\underset{i=1}{\sum }}\frac{v_{i}*MW_{i}}{\sigma_{i}*k_{cat,i}}\leq P_{total}*f$$

where $C^{T}$ is the transposed vector of the integer coefficient of each flux in the objective function Z; S is the stoichiometric matrix; lb and ub are the lower and upper bounds of the reaction fluxes in the system, respectively; $k_{cat,i}$ is the turnover number of enzymes that catalyze reaction i; $MW_{i}$ denotes the molecular weight of enzyme i;$\;\sigma_{i}$ is the saturation coefficient for enzyme i, whereby we use an average value of 0.5 for all the enzymes [18]; $P_{total}$ of 0.56 is the average protein content in most microbial cells [15]; f is the total mass fraction of all cellular enzymes in our ecGEM.

### 2.5. Calibration of the Original k_cat_ Values

Generally, the initial enzyme-constrained model was unable to accurately predict the experimental value of the maximal growth rate, requiring an adjustment of the original *k*_cat_ values, like GECKO, AutoPACMEN and ECMpy. Because no suitable 1^3^C data were found, the correction of ecCGL1 was performed using only method 1 proposed by ECMpy, which is based on enzyme usage. For enzymes that require calibration, the EC number was obtained and its corresponding *k*_cat_ value was substituted by the highest value in the BRENDA and SABIO-RK databases for the given enzyme class. This iterative correction process was continued until the experimental value or the predefined number of iterations was reached, as described in GECKO 2.0 (2.0 version) [19].

### 2.6. Comparative Flux Variability Analysis

We provided a fair comparison of flux variability range distributions between iCW773^R^ and ecCGL1 for experimental measurements of μ_max_ (e.g., 0.45 h^−1^ [33]), using the computational procedure of GECKO 2.0. For reactions containing isozymes, we used the maximal value of the corresponding flux variability range (Equation (6)). For reversible reactions from ecModel, the corresponding flux variability ranges were solved using Equation (7).

(6) $$FV_{i}=max\left(v_{i,iso_{j}}^{max}-v_{i,iso_{j}}^{min}\right),\;j\in m$$

(7) $$FV_{i}=\left(v_{i}^{max}-v_{i}^{min}\right)-\left(v_{i,REV}^{max}-v_{i,REV}^{min}\right)$$

### 2.7. Phenotype Phase Plane (PhPP) Analysis

The PhPP analysis is a powerful tool that provides a global view of how optimal growth rates are affected by changes in two environmental variables such as the carbon and oxygen uptake rate [34,35]. We performed PhPP analysis on iCW773^R^ and ecCGL1 to predict the biomass-specific growth rates by varying the glucose and oxygen uptake rates, as described in the literature [23]. Therefore, we varied the exchange reaction fluxes of glucose in the range of 0–10 mmol/gDCW/h and oxygen in the range of 0–10 mmol/gDCW/h, with the objective set to maximize the biomass production rate, after which the results were analyzed by parsimonious FBA (pFBA) calculations [36].

### 2.8. Simulation of Overflow Metabolism

We explored the overflow metabolism of *C. glutamicum* using ecCGL1 by varying the substrate uptake rate from 1 to 6.3 mmol/gDCW/h (the model reaches its maximum growth rate at 0.479 mmol/gDCW/h) and solving for the pFBA to maximize the biomass. To observe the trade-off phenomenon in unrestrained growth, we set glucose as the carbon source, and varied the substrate uptake rate from 1 to 6.3 mmol/gDCW/h to obtain the trade-off between the biomass yield (Equation (8)) and enzyme usage efficiency (Equation (9)). The $E_{min}$ value was calculated using the minimum enzyme amount algorithm of ECMpy [18].

(8) $$\mathrm{biomass}\;\mathrm{yield}=\frac{v_{biomass}}{v_{glucose}*MW_{glucose}}$$

(9) $$\mathrm{enzyme}\;\mathrm{usage}\;\mathrm{efficiency}=\frac{v_{biomass}}{E_{min}}$$

### 2.9. Prediction of Metabolic Engineering Targets

Enzyme cost can be used to identify key enzymes in the product synthesis pathway. For example, Ye et al. calculated the enzyme cost for l-lysine biosynthesis by fixing a low biomass growth rate (0.1 h^−1^) using the enzyme-constrained model of *E.coli* and improved l-lysine production by optimizing the expression of the proteins in the top 20 of the enzyme cost [22]. Because this approach can only identify overexpressed targets in the pathway, we extended it to explore enhanced and weakened metabolic engineering targets. First, we determined the protein cost differences in reactions between two scenarios: high growth low product generation (HGLP, growth rate was set at 0.46 h^−1^) and low growth high product generation (LGHP, growth rate set at 0.1 h^−1^). Subsequently, we calculated the cost of each reaction (Equation (10)) for both pathways [18]. Finally, we calculated the fold changes of enzyme cost and those with a fold change great than 1.5 were chosen as potential targets for metabolic engineering (Equations (11) and (12)).

(10) $$Enzyme\;cost_{i}=\frac{v_{i}*MW_{i}}{\sigma_{i}*k_{cat,i}}$$

(11) $$Enhance\;target=\left\{Enzyme|\frac{Enzyme\;cost_{LGHP}}{Enzyme\;cost_{HGLP}}\geq 1.5\right\}$$

(12) $$Weaken\;target=\left\{Enzyme|\frac{Enzyme\;cost_{HGLP}}{Enzyme\;cost_{LGHP}}\geq 1.5\right\}$$

## 3. Results

### 3.1. GPR Correction of iCW773

There are 1207 reactions in the iCW773 model, 96 of which are “and” relationships. We obtained protein composition information for a total of 112 reactions using the GPRuler tool, of which 24 had GPR relationships consistent with the model (Table S3). The 88 reactions with inconsistent GPR relationships were manual checked and corrected using information from databases like UniProt, BioCyc, KEGG and the literature (Table S3). They can be classified into three categories. First, the GPR relationship in the model is correct, so there is no need to replace (14 reactions). Second, the prediction results of GPRuler tool are correct and can be used to replace the GPR relationship in the model directly (12 reactions). For example, the GPR relationship of succinyl-CoA synthetase is ‘*Cgl2565* or *Cgl2566*’ in the model. However, in UniProt, Succinyl-CoA synthetase is described as a heterotetramer containing two alpha and two beta subunits. The beta subunit (*Cgl2566*) provides nucleotide specificity of the enzyme and binds the substrate succinate, while the binding sites for coenzyme A and phosphate are found in the alpha subunit (*Cgl2565*). Third, both the prediction results of the GPRuler tool and the GPR relationship in the model are wrong and should be manual corrected using Uniprot, BioCyc, KEGG or the literature (62 reactions). For example, the gene composition of the Adenosylcobalamin 5′-phosphate synthase (ADOCBLS) is given as ‘*Cgl0245* and *Cgl2201*’ in the model, while from the GPRuler tool, the GPR relationship were obtained as ‘*Cgl0245* and *Cgl0246*′. Whereas *Cgl0245* and *Cgl0246* do form a protein complex, its function is Lipid II isoglutaminyl synthase (glutamine-hydrolyzing) rather than Adenosylcobalamin 5′-phosphate synthase. Therefore, we corrected the GPR relationship for this reaction to ‘*Cgl2201*′ as *Cgl2201* is the true adenosylcobalamin-5′-phosphate synthase based on information from UniProt.

In addition, we observed that 58 “and” relationships in the model were not identified by the GPRuler tool. To determine the correctness of these relationships, we calculated the protein sequence similarity for all gene pairs which have ‘and’ relationships in the model. The results showed no similarity between protein pairs in the 41 reactions (Table S4). However, the remaining 17 reactions with an ‘and’ relationship have high similarities (great than 20%) between the corresponding proteins, which are more likely to be isozymes. For example, the GPR relationship for Methylisocitrate lyase (MCITL2) is ‘*Cgl0658* and *Cgl0695*’, which has 81% sequence similarity. By further searching the KEGG database for verification, we found that *Cgl0695* and *Cgl0658* have the same protein name, catalyse the same reaction and map the same EC number, but not any information implying they are subunits of a protein complex. They are more likely to be two isoenzymes and, therefore, the correct GPR relationship for Methylisocitrate lyase should be ‘*Cgl0658* or *Cgl0695*’. After manual investigation, we modified ‘and’ relationships to ‘or’ relationships for 17 reactions in the model (Table S4). Finally, the new model contains 1194 reactions and 794 genes, which we have named iCW773^R^ (Revised iCW773).

### 3.2. EcModel Calibration

After that, we split each reversible reaction into independent forward and reverse reactions, including 333 reactions. The isozyme-catalyzed reactions were divided into multiple reactions (append num in reaction ID, e.g., ACOATA_num1), and a total of 202 reactions were split into 571 reactions. After this step, the number of reactions in the model was expanded to 1850. The growth rate predicted by the initial ecModel at a glucose uptake rate of 5.05 mmol/gDCW/h was low compared to the experimental value (0.12 h^−1^ vs. 0.45 h^−1^) [33]. We calibrated the initial ecModel based on the enzyme usage, and after 13 rounds of calibration and modification of 10 reactions, the simulated growth rate of 0.423 h^−1^ was close to the experimental value (Table S5). Although the growth rate was corrected, the abnormal flux in the TCA cycle was mainly caused by the exceptionally large molecular weight of the pyruvate dehydrogenase complex consisting of 24 subunits, which was corrected using the maximal *k*_cat_ of this enzyme in the databases (Figure S2). After this modification, the growth rate reached 0.454 h^−1^, which was consistent with the experimental value.

### 3.3. Basic Information of ecCGL1

**Figure 2:**
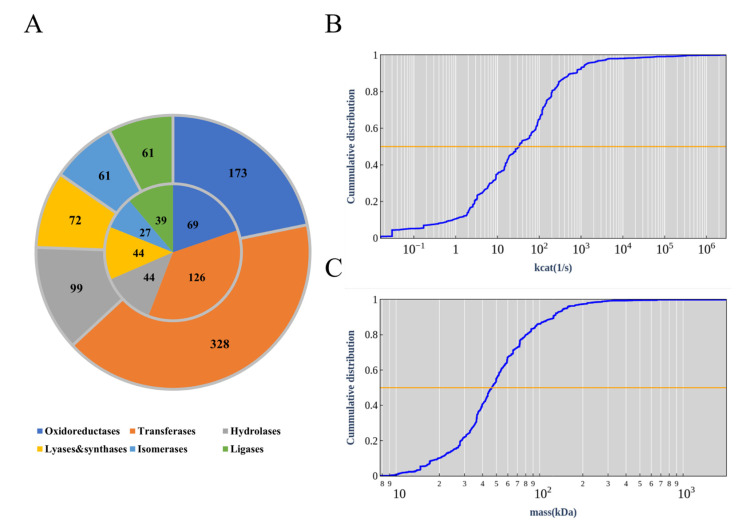
Basic information of ecCGL1. (**A**) Enzyme classification. The outer ring shows the 646 reactions with available enzyme kinetic data, which were divided into six categories. The inner ring shows the 304 different enzymes that catalyze these reactions according to the EC numbers, which can also be divided into six categories. (**B**) Cumulative distribution of *k*_cat_ values. (**C**) Cumulative distribution of molecular weights.

There were 794 reactions in the modified ecModel with available *k*_cat_ data of enzymes with EC numbers, accounting for 42.92% of the total reactions, which were divided into six major categories, most of which were transferases (Figure 2A, outer ring). These 794 reactions were catalyzed by a total of 349 enzymes (different EC numbers) (Figure 2A, inner ring), and the *k*_cat_ values spanned seven orders of magnitude with a median value of 33.3 s^−1^ (Figure 2B). There are 1107 enzymes present in the ecCGL1, while non-monomeric enzymes occupy 30.81% (Figure S3). The distribution of subunit composition in *C. glutamicum* is slightly different to that of *E. coli* [18], in that the proteins containing more than two subunits are generally low in *C. glutamicum* (Figure S3). The biological reason for the difference is not clear and might be an interesting topic for further research. Finally, the molecular weights of the enzymes spanned a range from 7 to 2000 kDa (Figure 2C).

As the protein abundance of *C. glutamicum* could not be found in PAXdb [37], we obtained the abundance of each protein from the published data [31,32]. An f value of 0.46 was obtained by calculating the mass fraction of enzymes that could be expressed by the genes in the model, which was a representation of a percentage of the total protein constraints in *C. glutamicum*.

### 3.4. EcCGL1 Reduces the Solution Space

**Figure 3:**
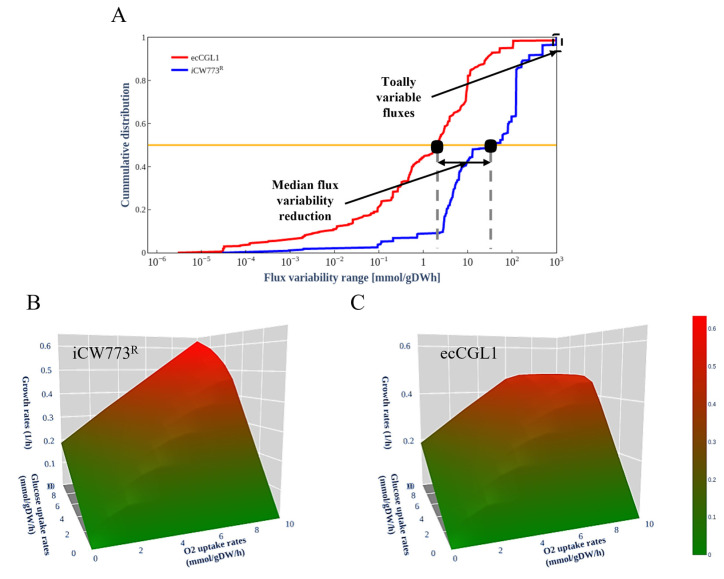
The solution space of iCW773^R^ and ecCGL1. (**A**) Cumulative distribution of flux variability of ecCGL1 and iCW773^R^ at high growth rates. Growth rates at various glucose and oxygen uptake rates were simulated using iCW773^R^ (**B**) and ecCGL1 (**C**).

A major challenge for GEMs is how to obtain a biologically meaningful flux distribution, as there are alternate optimal solutions in which the same maximal objective can be achieved through different flux distributions [38]. This limitation can be overcome by integrating experimentally measured exchange fluxes as constraints [19]. Previous studies have demonstrated that enzyme constraint models can significantly reduce the solution space of fluxes [18,19]. We compared the cumulative distributions of the flux variability ranges of iCW773^R^ and ecCGL1, which revealed that the median flux variability range at a high growth rate is significantly reduced by 1 orders of magnitude after introducing enzyme constraints (Figure 3A). The cumulative distribution also showed a decrease in the number of reactions with completely variable fluxes, which may represent undesirable futile cycles caused by a lack of information regarding their thermodynamic or enzymatic costs [19]. At high growth rates, completely variable fluxes accounted for 4% of the active reactions in iCW773^R^, in contrast to the complete absence of this extreme range of variability in ecCGL1.

With the subsequent increase of the carbon source consumption and oxygen fluxes, PhPP analysis showed that the growth rate of iCW773^R^ increased linearly with increasing carbon source consumption, which is inconsistent with the experimental observations, while ecCGL1 significantly reduced the solution space (Figure 3B,C). All these results demonstrate that incorporating more information and constraints into a GEM can improve the predictive accuracy of the model and enable it to simulate a more realistic cellular phenotype.

### 3.5. Simulation of Overflow Metabolism

**Figure 4:**
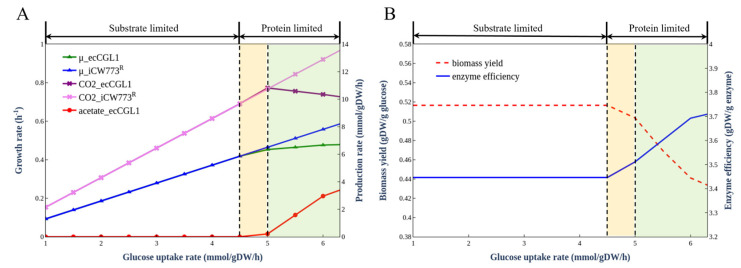
Simulation of overflow metabolism. (**A**) Comparison of in silico overflow metabolism between iCW773^R^ and ecCGL1. (**B**) Trade-off phenomenon simulated by ecCGL1.

In previous studies, ecModels were used to simulate overflow metabolism in *S. cerevisiae* [16] and *E.coli* [18]. As shown in Figure 4A, ecCGL1 was also able to precisely simulate the overflow metabolism phenomenon at a glucose uptake rate of 4.5 mmol/gDCW/h, which could not be reproduced in iCW773^R^. When overflow metabolism occurs, the microorganism must activate a fermentation pathway with low energy production efficiency but high enzyme efficiency to maintain growth, and this pathway will cause a portion of the substrate to be converted into by-products [39], resulting in carbon loss and a rapid decrease of the biomass yield, illustrating a trade-off between enzyme efficiency and growth rate (Figure 4B). This metabolic process can be divided into a substrate-limited stage, overflow switching stage, and overflow stage. In the first stage, the glucose uptake rate is low (less than 4.5 mmol/gDCW/h) and has a linear relationship with the growth rate, which is consistent with iCW773^R^ (Figure 4A). When the substrate supply is gradually increased (between 4.5 and 5 mmol/gDCW/h), the cell growth is limited, and metabolism is switched to a more enzymatically efficient pathway. Finally, when the overflow metabolism phenomenon occurs (greater than 5 mmol/gDCW/h), the by-product pathway of acetate production switches to higher enzymatic efficiency, consuming more substrates and resulting in lower biomass yield, which is consistent with empirical models of microbial growth [40].

### 3.6. Exploration of the Targets Based on Enzyme Cost

**Figure 5:**
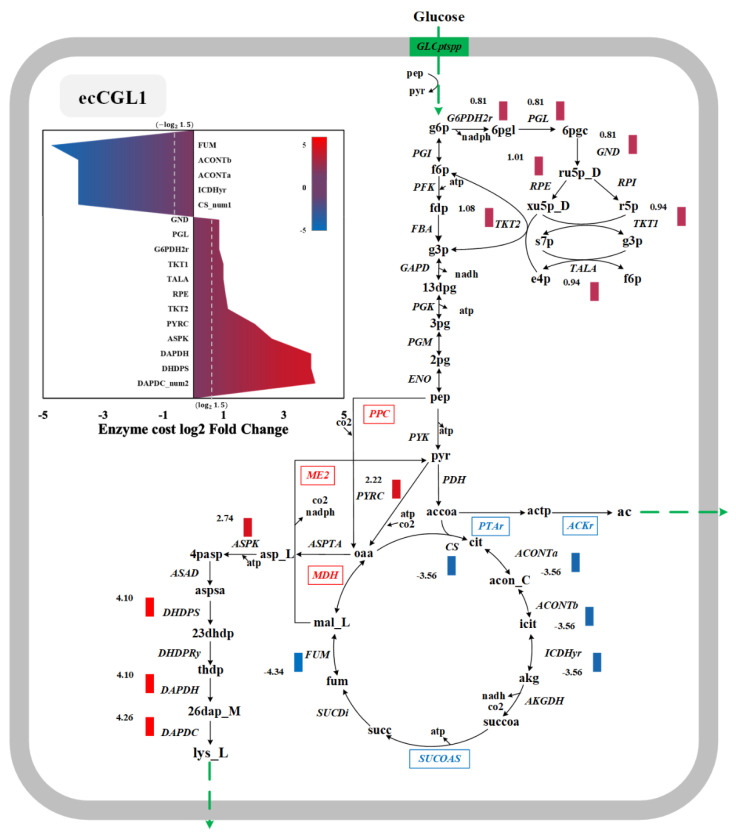
Simulation of differential pathways under high and low l-lysine production conditions. The red box indicates reactions that are more beneficial for l-lysine production, and the blue box indicates reactions that are more beneficial for growth. Red bars indicate enhanced targets for l-lysine production in *C. glutamicum*. Blue bars indicate weak targets that may increase l-lysine production in *C. glutamicum*. Green lines indicate the movement of compounds in and out of the cell membrane.

We compared the pathway characteristics at LGHP and HGLP to analyze the differences in the product synthesis pathways and found 23 reactions in which the change of enzyme cost was higher (Figure 5). We first noticed the pathway changes in the l-lysine synthesis pathway between the two conditions, some reactions (e.g., ME2, PPC and MDH) were more favorable for l-lysine production (with increased flux) and some others (e.g., PTAr, ACKr and SUCOAS) are in contrast (Figure 5 red and blue boxes). For example, malate dehydrogenase (ME2) catalyzes the conversion of malate to pyruvate to regenerate pyruvate depleted by pyruvate carboxylase (PYRC) [41]. The PYRC catalyzed the conversion of pyruvate to oxaloacetate, a known precursor of l-lysine synthesis [40]. Therefore, ME2, MDH and PYRC, form a cycle for NADPH regeneration, which can provide more NADPH for the l-lysine synthesis pathway. Furthermore, the pyruvate-oxaloacetate (OAA) supply has been considered a bottleneck for l-lysine production, while overexpression and point mutation of phosphoenolpyruvate carboxylase (PPC) has been applied to increase OAA availability [42,43,44].

At the same time, some reactions were more amenable for high-level growth and should be downregulated for l-lysine production. For example, decreased flux via succinyl-CoA synthetase (SUCOAS) was reported to increase the l-lysine yield and maintain optimal cell growth at the same time [45]. In addition, as we mentioned above, when enzyme constraints occurred, the acetate overflow phenomenon can be captured by ecCGL1. It was reported that the production of organic acids such as acetate or lactate may reduce the yield of biological products [39]. Consequently, less flux toward acetate overflow reactions (PTAr and ACKr) may result in more l-lysine production.

After analyzing these pathway changes, we further analyzed the enzyme costs of the l-lysine synthetic pathway calculated from the enzyme-constrained model. To explore the variability of enzyme costs, we calculated the log2-fold changes of enzyme costs between LGHP and HGLP, as shown in Figure 5. We found that the upregulated values were mainly focused on the pathway of l-lysine synthesis from aspartate (DAPDC, DAPDH, DHSPS, etc.) and the pentose phosphate (PP) pathway (TKT1, TALA, GND, etc.), which was in agreement with the literature [41,46,47]. In addition, lower fluxes in TCA may lead to more fluxes for lysine production [48,49,50]. For example, Jan et al. showed that reduced citrate synthase (CS) activity leads to a strong accumulation of l-lysine [50].

## 4. Discussion

We constructed the enzyme-constrained model ecCGL1 based on the iCW773 model of *C. glutamicum* using the upgraded workflow (Figure 1). First, we updated the workflow of ECMpy to automatically acquire *k*_cat_ values from databases and fill missing values using AutoPACMEN. In the construction of the ecModel of *E. coli* using ECMpy, we have emphasized the impact of the subunit composition of proteins on the accuracy of model simulations, but it was done by manually correcting GPR relationships and collecting the subunit number of each protein [18]. In this study, we achieved the semi-automated correction of GPR relationships using the GPRuler tool and protein homology similarity, and the automated acquisition of the quantitative subunit composition data based on UniProt. The GEMs and most of the ecGEMs neglect the quantitative subunit composition information of non-monomeric enzymes, so the introduction of the number of subunits in the GPR relationship might also be necessary for future model reconstruction.

The ecCGL1 model exhibited a better simulation accuracy of strain behavior than the original iCW773. The growth rate increased linearly with the substrate uptake in the classical model, while the enzyme constraint narrowed the solution space, resulting in a model prediction that is closer to the real experimental observations. Due to growth rate limitations and enzyme resource constraints, ecCGL1 was better able to predict the phenomenon of overflow metabolism, which was absent from the original model. This indicates that enzyme restriction is the primary driver behind enzyme protein redistribution and corresponding metabolic flux changes, which was consistent with previous studies [51]. Consequently, our study not only corroborates the hypothesis that effective proteome reassignment is an important principle of metabolic regulation, but also shows how simple physicochemical constraints can be integrated into a GEM to improve its predictive power. Our model also made predictions based on the enzyme cost, offering a more intuitive reproduction of metabolic engineering strategies than the original GEM. According to the results of simulations using ecCGL1, some potential targets in the glycolysis pathway should be considered for improving l-lysine production in the future, which may generate more energy and phosphoenolpyruvate, thus redistributing more flux toward the l-lysine synthesis pathway and cell growth.

Even though the ecCGL1 model offers better phenotype predictions, it still has several shortcomings. First, the GEM of *C. glutamicum* is still evolving and there is still a lack of annotated information in some areas. Recently, Feierabend et al. reconstructed the iCGB21FR model using iEZ482 as a reference [52], which added seventeen different databases that are cross-referenced in the model’s annotations and reached a high MEMOTE score (Figure S1) [53]. This new model expands the number of reactions in the GEM of *C. glutamicum* to 1892. Of course, our process for constructing the enzyme-constrained model of *C. glutamicum* is generic, and another enzyme-constrained model can be constructed by simply replacing the initial model. Besides, the quality of the enzyme-constrained model depends on the input data on enzyme kinetics and intracellular protein abundance [16]. Unfortunately, there is little data on both for *C. glutamicum*. There are three ways to improve the coverage of enzyme kinetic parameters in the model: (1) directly populate unknown reactions with mean or median values of enzyme kinetic parameters from other reactions [16,17,18]; (2) expand the EC number annotation information of model reactions using EC number prediction tools [54,55]; and (3) directly predict reactions with unknown parameters based on existing enzyme kinetic parameters via machine learning or deep learning approaches [25,56]. In addition, the kinetic data were mainly sourced from the BRENDA and SABIO-RK databases, which mostly collect in vitro measurements that differ somewhat from the in vivo data. The improvement of parameter accuracy and coverage will increase the prediction efficiency and reduce the cost of result evaluation, which will help construct high-quality metabolic models of species such as *C. glutamicum*. Finally, although ecGEM has improved predictive power compared to traditional GEMs, biological systems are also subject to other constraints in addition to enzyme resources, and the construction of multi-constraint models (e.g., ETGEM [57] and ETFL [58]) will certainly provide new prospects for systems biology research.

## 5. Conclusions

In this study, we constructed an enzyme-constrained genome-scale metabolic model of *C. glutamicum* (ecCGL1) by integrating various enzymatic parameters at the entire network level. The results show that constraints on enzyme resources can simulate strain growth limitations and recapitulate metabolic overflow phenomena, resulting in more realistic pathway predictions, which can be used to identify key enzymes to provide metabolic engineering targets for creating cell factories to produce valuable chemicals.

## Data Availability

The scripts and datasets generated and/or analyzed in the study can be found at: https://github.com/tibbdc/ecCGL1, accessed on 30 August 2022.

## Supplementary Materials

The following supporting information can be downloaded at https://www.mdpi.com/article/10.3390/biom12101499/s1. Figure S1: Model development of *Corynebacterium glutamicum;* Figure S2: Pathway changes after changing the *k*_cat_ of PDH; Figure S3: Number of subunits per complex (only consider complex with two or more subunits) in *C. glutamicum*; Table S1: Keywords of protein complex formation in UniProt; Table S2: Correspondence between subunit descriptions and true numbers in UniProt; Table S3: GPR modifications based on GPRuler tool results; Table S4: GPR Modifications by homology similarity; Table S5: Reactions for *k*_cat_ calibration.
