# Supplementary figures and images for: Construction and Analysis of an Enzyme-Constrained Metabolic Model of Corynebacterium glutamicum
Source: Biomolecules. 2022 Oct 17;12(10):1499. doi: 10.3390/biom12101499 (PMC9599660; doi:10.3390/biom12101499)

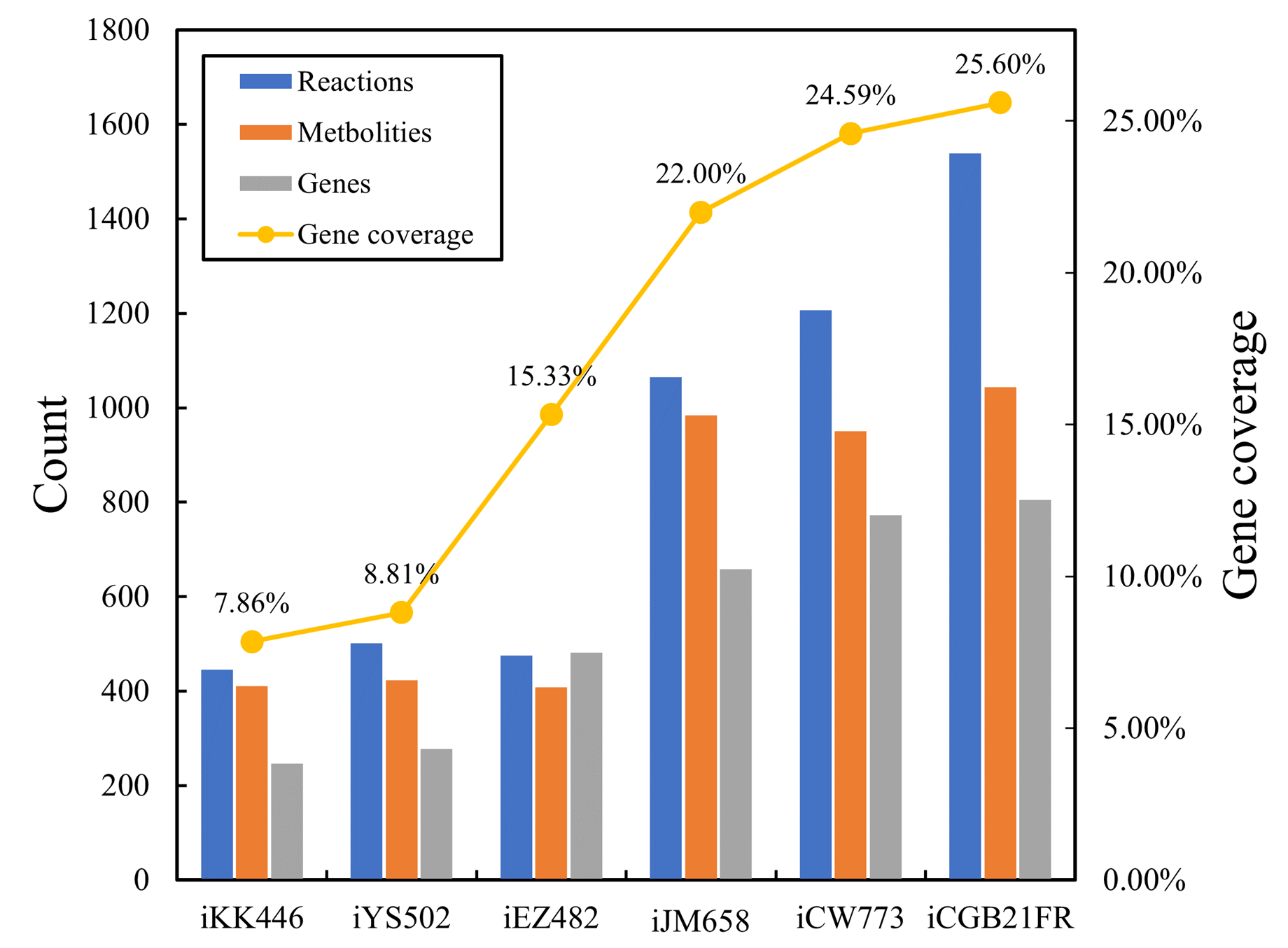

Supplement: Supplementary file 1 [file biomolecules-12-01499-s001.zip › Figure S1.tif]

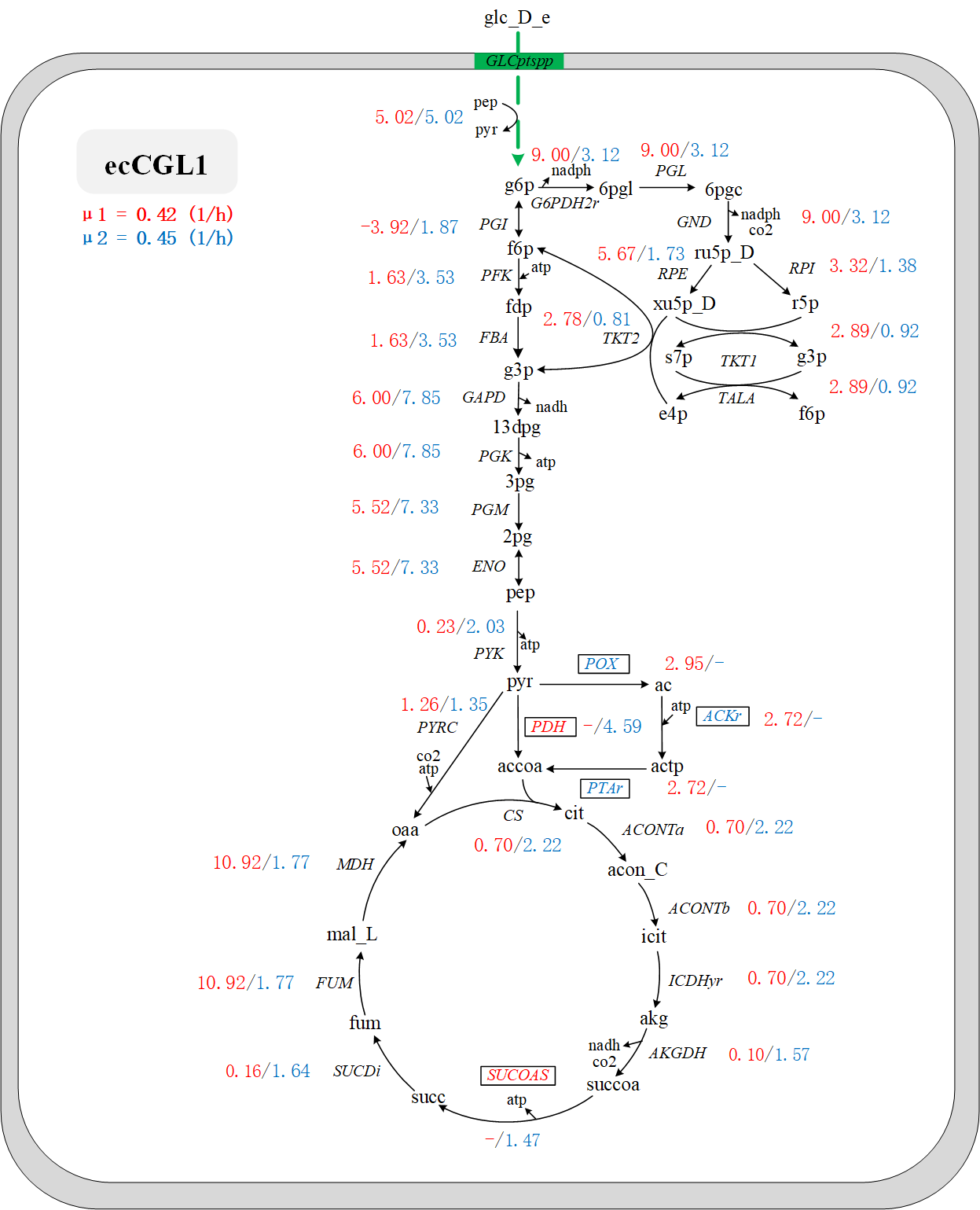

Supplement: Supplementary file 1 [file biomolecules-12-01499-s001.zip › Figure S2.tif]

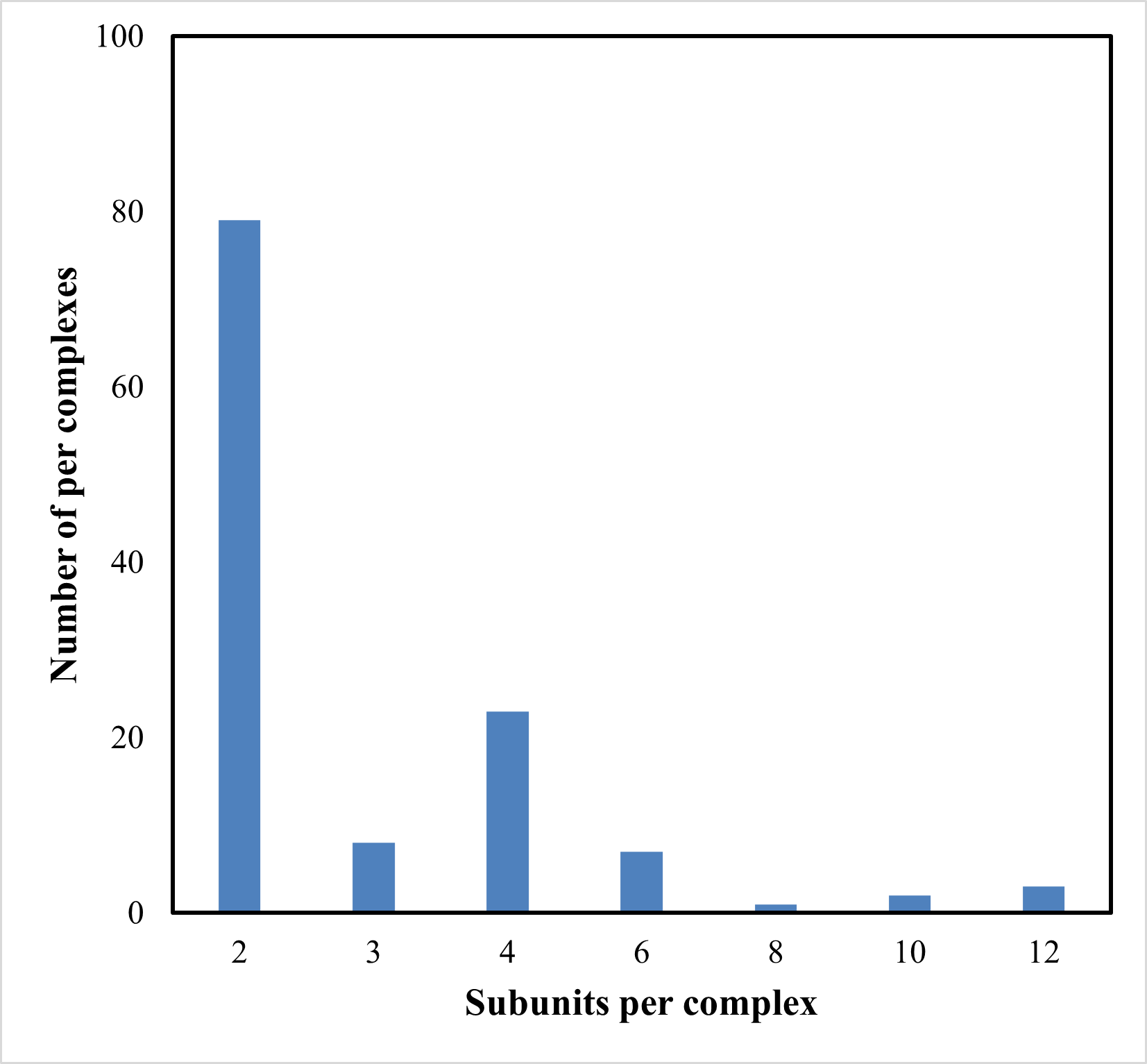

Supplement: Supplementary file 1 [file biomolecules-12-01499-s001.zip › Figure S3.tif]
